# Nurses' perception, knowledge, and use of neonatal pain assessment

**DOI:** 10.1002/pne2.12050

**Published:** 2021-05-07

**Authors:** Martina Carlsen Misic, Randi Dovland Andersen, Sofia Strand, Mats Eriksson, Emma Olsson

**Affiliations:** ^1^ Department of Pediatrics Faculty of Medicine and Health School of Health Sciences Örebro University Örebro Sweden; ^2^ Faculty of Medicine and Health School of Health Sciences Örebro University Örebro Sweden; ^3^ Department of Research Telemark Hospital Trust Skien Norway

**Keywords:** neonatal pain assessment, neonatal pain management, newborn infant, nursing

## Abstract

Preterm and sick newborn infants undergo several painful procedures during their hospital stay, potentially leading to short‐ and long‐term negative consequences. Pain assessment should be performed regularly to provide optimal pain management. Nurses' knowledge of and attitude toward neonatal pain assessment affect how pain is assessed and managed in the clinical situation. The aim of this study was to explore Swedish nurses' perception, knowledge, and use of neonatal pain assessment. This descriptive, cross‐sectional questionnaire study was conducted across all Swedish neonatal units (n = 38). Respondents were chosen through convenience sampling by the head nurses at each unit. Ten nurses from each unit were asked to complete the survey, which contained both closed and open questions. A majority of the units (30/38; 79%) participated and 232 surveys were returned, a response rate of 61%. Of the nurses, 91% thought that neonatal pain assessment was important. Many nurses mentioned various difficulties with pain assessment and concerns that the scales used might not assess pain correctly. About half of the nurses considered themselves to have enough knowledge of neonatal pain assessment. Those who reported having enough knowledge of pain assessment viewed the pain scales used at their units more positively. Of the nurses, 74% reported using a pain assessment scale several times per work shift. Pain management guidelines were available according to 75% of nurses, but only 53% reported that the guidelines were followed. Although nurses in general expressed a positive attitude toward pain assessment scales, this was not necessarily evident in their clinical practice. Lack of knowledge, available or accessible guidelines, or concerns regarding the validity of available pain scales seemed to limit their use.

## 1 BACKGROUND

About 10% of newborn infants need neonatal care, and during their hospital stay, they are exposed to several painful procedures.^1^, ^2^ Repeated painful experiences and stress in the newborn period can lead to increased pain sensitivity, altered pain behavior, and poor cognitive outcomes later in life.^3^ To minimize these negative effects and ensure treatment of pain, regular pain assessment is recommended.^4^ There is still no objective way to assess pain in the daily clinical work in neonatal units, and professionals assess newborn infants' pain using various pain scales based on structured observation of pain‐related behaviors.^5^ Pain assessment should be performed, documented, and evaluated with the same regularity as other vital parameters.^4^, ^6^, ^7^ To provide the newborn infant with adequate pain treatment, it is important to acknowledge pain and how the infant expresses it.^8^

In accordance with national guidelines for pain management in newborn infants, the professionals who are working closely with the infant, most commonly nurses, are responsible for the assessment and documentation of pain. To prevent pain, the assessment should also lead to adequate actions to prevent and treat pain, for example, optimized environment, behavioral support, or pharmacological treatment.^9^ Despite the importance of treating pain in newborn infants, many newborns' pain is still under‐assessed and undertreated. A multicentre study in Europe showed that only 10% of preterm infants received daily pain assessment for ongoing pain and that two thirds of intubated babies never received any pain assessment at all.^1^

There has been an increase in systematic pain assessment in neonatal care in Sweden over the last 15 years,^10^ and most (86.6%) head nurses in Swedish neonatal units reported that pain assessment was routine and that a validated pain scale was used.^11^

Various matters can affect how nurses assess pain. When nurses' pain assessment does not lead to adequate interventions, nurses become discouraged from continuing pain assessment.^12^ A lack of trust in the pain scale used can cause nurses not to use the scale clinically.^13^ Other obstacles to adequate pain assessment can be issues of communication between different professions, lack of knowledge, and fear of side effects from pharmacological treatments.^14^ It is therefore important to understand how nurses perceive their knowledge of, attitudes toward, and use of neonatal pain assessment in their daily clinical work. Several studies have been carried out, concerning compliance with pain guidelines. Many of these previous studies were either knowledge and opinion surveys about pain assessment,^7^, ^8^ or surveys to the head nurses,^10^, ^11^ whereas less is known about the views and practices of the clinically working nurses. Despite that the uptake of guidelines seems to have improved, there is still a significant knowledge‐practice gap and therefore necessary to monitor the development in clinical use of pain assessment and management on a regular basis.

## 2 METHODS

The aim of the study was to explore Swedish neonatal nurses' perception, knowledge, and use of neonatal pain assessment.

### 2.1 Design and setting

This descriptive, cross‐sectional study distributed questionnaires to all Swedish neonatal units study was distributed to all Swedish neonatal units (n = 38).

#### 2.1.1 Respondents

The respondents were a convenience sample of up to 10 nurses, specialist nurses, or midwives from each participating unit, for a total of 380 possible respondents. The midwives included were working as nurses in the neonatal units and are therefore referred to as nurses in the result. Each head nurse was asked to select respondents with a range of neonatal experience working clinically at the unit. Other groups of staff such as assistant nurses, doctors, psychologists, and physiotherapists were excluded due to the aim of this study. Other exclusion criteria were students and new nurses. One invited unit was excluded from the survey after the head nurse explained that they only cared for healthy newborn infants, as the unit was a maternity ward.

#### 2.1.2 Questionnaire

Data were collected in spring 2018. The questionnaire was developed by the authors and consisted of 22 open and closed questions related to the aim of the study. The closed questions were answered on a five‐point Likert scale, while the open questions allowed respondents to clarify their answers to the closed questions. Examples of questions in the questionnaire are: “How important do you think neonatal pain assessment is?” and “How good is your knowledge of neonatal pain assessment?” The questionnaire was pilot tested with two nurses and one assistant nurse and resulted in only minor changes (Appendix S1).

#### 2.1.3 Data collection

The survey was mailed to the head nurses at each neonatal unit together with information about the study and prepaid return envelopes. Each survey contained information about the study and stated that the participant consented to participate by completing and returning the questionnaire. According to Swedish law, for an anonymous survey of health care personnel ethical review and approval are not needed. Participation was voluntary and anonymous as no personal data were collected. The respondents were initially given 14 days to complete the surveys, and the head nurses were asked to collect and return the surveys by mail. Reminders were sent out 2 and 4 weeks after the elapsed answering time.

#### 2.1.4 Data analysis

The data were analyzed using descriptive statistics, and to present and analyze differences between units and respondents, Chi‐square testing, Kruskal‐Wallis testing, and one‐way ANOVA were used. The level of significance was set to *P* < .05. Data were analyzed using SPSS version 24.0 for Windows (IBM Corp.) and Excel 2016 (Microsoft Inc). Quotations from the open answers were sorted into matching categories, and these answers were used to illustrate the findings from the closed questions. Examples of categories found in the responses to the open question “Why are guidelines not followed?” were lack of knowledge and lack of trust in the pain scale used at the unit.

## 3 RESULTS

Of all neonatal units in Sweden, 79% (n = 30) participated and 61% (n = 231) of the surveys were completed. The neonatal units were classified by the type of specialized care they provided. Most units that responded were level 3a units (n = 20, 69%), while the remaining units were either level 1 (n = 2, 7%), level 2 (n = 2, 7%), or level 3b (n = 5, 17%) units.^15^

Ninety‐nine percent of the respondents were women (n = 229). Respondents were between 22 and 65 years old and had 0‐42 years of neonatal care experience. Of the respondents, 67% had a specialist nursing education. See Table 1 for the demographic data.

**TABLE 1:** Demographic data of the participating nurses

Gender, n (%)	
Women	229 (99)
Men	2 (1)
Age, mean (stdv)	40.1 (11.3)
Years working within neonatal care, mean (stdv)	11.6 (11.1)
Specialist nurse education, n (%)	
Yes	156 (68)
No	75 (32)
Number of respondents per unit	
Min	3
Average	8
Max	10

### 3.1 Nurses' perception of neonatal pain assessment

Almost all the nurses (99%) answered that pain assessment was important. However, only 54% answered that the infants' care improved greatly with the use of pain assessment scales and 3% thought that the infants' care was improved only minimally with the use of pain assessments. Almost all respondents (92%) noted that a combination of a validated pain scale, physiological measures, and clinical appearance was the best way to observe pain signals in infants. Five percent thought that the clinical sense alone was the best way to recognize the infants' pain signals. If an infant received a high score according to the pain assessment scale, 71% of the nurses thought that the infant would receive pain‐relieving treatment in some or most cases. Most nurses (72%) experienced difficulties with pain assessment, and one nurse commented “It's not always that easy, sometimes it is very difficult”. The most commonly identified difficulties with pain assessment were lack of trust in the pain scale, lack of pain assessment routines, and difficulties in interpreting the infants' signals.

#### 3.1.1 Lack of trust in the pain scale

Many nurses mentioned that the pain scale was not objective and that the pain score the infant received could differ depending on the individual performing the assessment: “Because we conduct the observation from our own experience, it could be interpreted differently.”

Several nurses noted that an infant sometimes received a high pain score even though pain was not the main issue for the infant: “You need a lot of practice to recognize pain signals. The same signals could be from totally different reasons.” Some of the nurses also described difficulties assessing pain in sedated or severely ill infants who were unable to express their pain: “It is sometimes hard to determine whether the infant is relaxed or listless.” The nurses also mentioned that it was hard to assess the infants when they were covered in blankets.

#### 3.1.2 Lack of routines

Several nurses thought that the pain management guidelines in their units were unclear and that pain assessment was not implemented routinely in their unit. This prevented the use of pain assessment in the daily work. Lack of knowledge and experience could also limit correctly performed pain assessments: “There is a lack of knowledge among the staff, and pain assessment is often given a low priority. Instead, you go with your feeling.”

#### 3.1.3 Poor collaboration with doctors

Some nurses also mentioned poor co‐operation with the physicians as a problem. Sometimes when an infant reached a high pain score in the assessment, the physician did not agree or was not willing to prescribe pharmacological treatment due to fear of side effects.

### 3.2 Nurses' knowledge of pain management and assessment

About half of the responding nurses (63%) thought they had enough or more than enough knowledge of neonatal pain assessment, and nurses with a specialized nursing education were more likely to consider that they had enough knowledge (*P* < .001).

**FIGURE 1:**
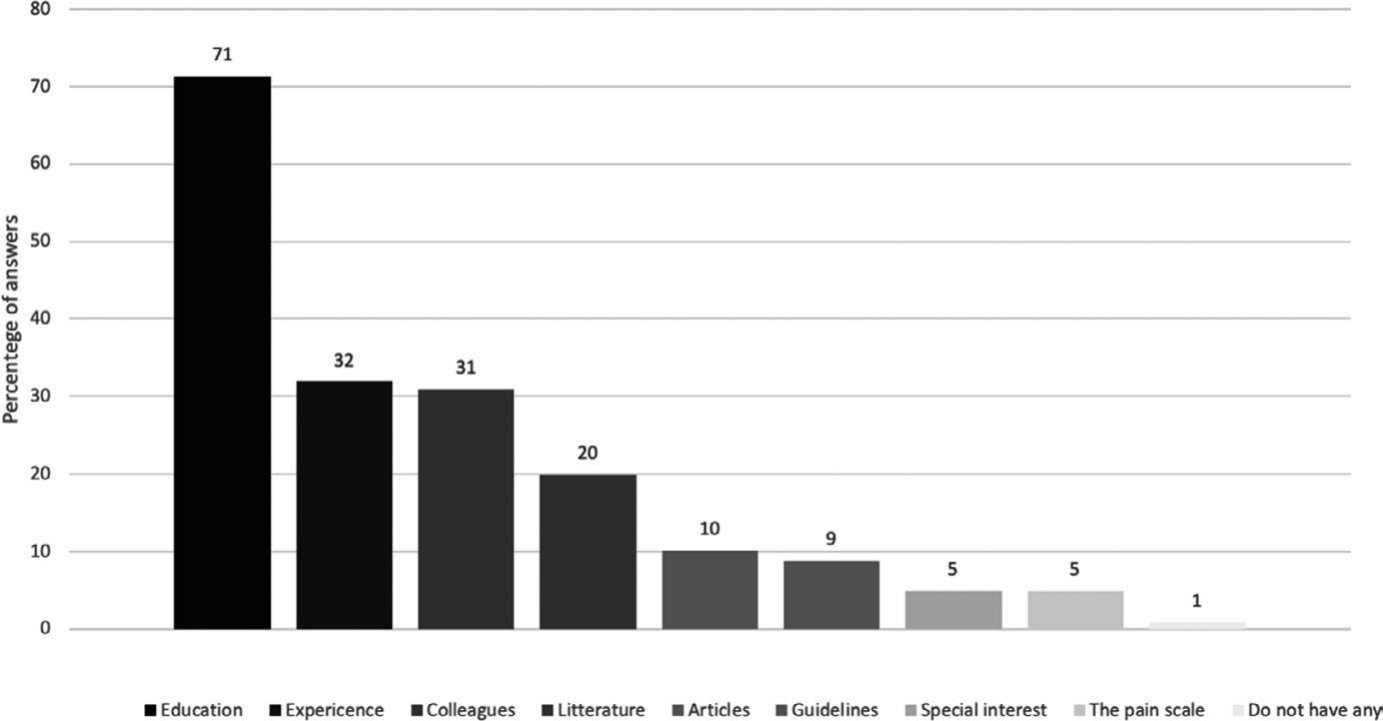
Sources of knowledge about neonatal pain assessment. Absolute numbers, more than one answer was possible

The most common way to gain knowledge of neonatal pain assessment was through education (71%), either at their unit or during their specialist education. Other ways to gain such knowledge were through clinical experience (31%) and from colleagues (29%) (Figure 1). Of the respondents, 66% thought that they had enough knowledge to use a pain scale correctly, 20% felt insecure, and 14% thought they did not have enough knowledge. Nurses who answered that they had enough knowledge of neonatal pain assessment also thought they had enough knowledge to use the pain scale correctly (*P* ≤ .001).

### 3.3 Nurses' use of pain assessment scales

**FIGURE 2:**
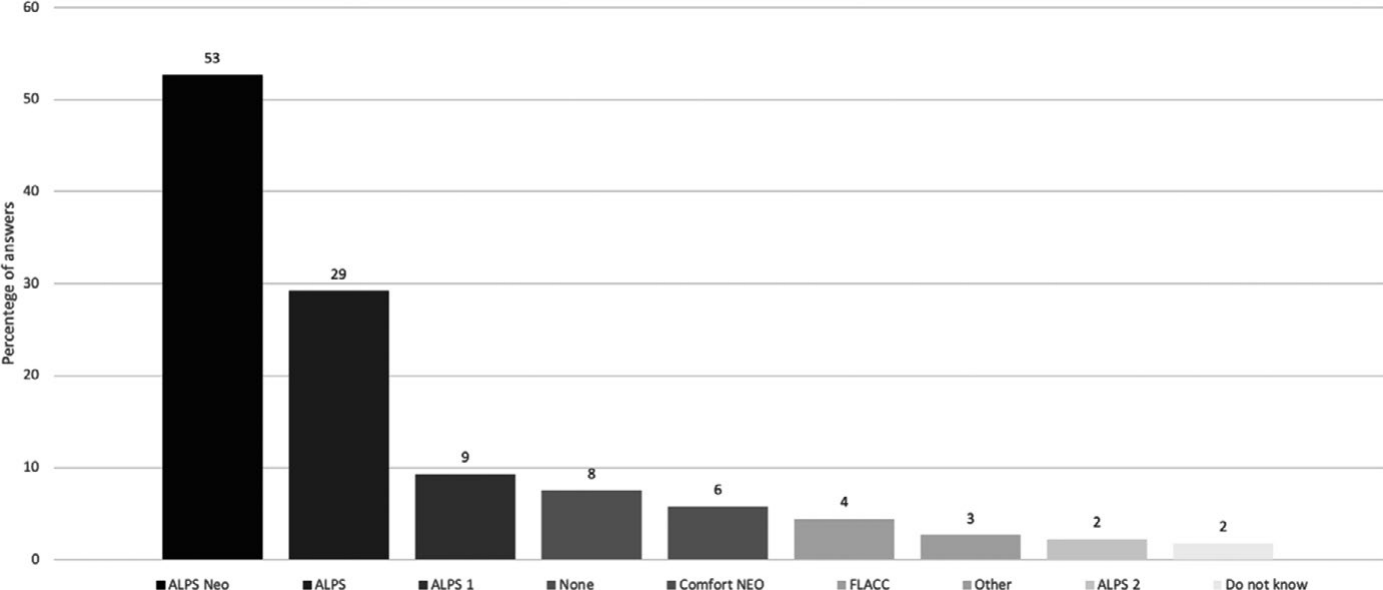
Pain scales used by the nurses. Absolute numbers, more than one answer was possible

The pain scale most commonly used by the participating nurses (51%) was the Astrid Lindgren and Lund Children's Hospital Pain Scale (ALPS‐neo), ALPS‐neo is one of several different versions of the ALPS scale, and 28% answered that they used ALPS scale in some form but did not specify which version they used (Figure 2). Eight percent of the nurses answered that no scales were used in their unit. Most of the nurses answering that no scale was in use were from one of three same units.

Attitudes toward the pain scales used in the neonatal units were mostly positive or very positive (72%). Nurses who reported that they had enough knowledge of pain assessment viewed the pain scale they used more positively (*P* = .001). Furthermore, the nurses who thought they had enough knowledge on how to use the pain scale correctly also viewed the pain scale more positively (*P* < .001) (Table 2). Three quarters of the nurses (74%) reported that they used a pain scale regularly in their work, and 32% used the scale several times during a work shift. Some nurses concluded that the newborn infants themselves determined how many times they assessed pain during a shift. Most nurses (77%) reported that pain was assessed more frequently in infants needing intensive care than in infants receiving a lower level of care. In the open questions, the nurses described how infants cared for in family rooms did not receive pain assessments since these infants were not as sick as the infants in intensive care.

**TABLE 2:** Knowledge of the pain scale in use in relation to attitude toward the scale

	Attitude toward the pain scale in use	
	Negative	Positive
Knowledge on how to use the scale		
Yes	28 (19)%	119 (81)%
No	13 (54)%	11 (46)%
Unsure	18 (45)%	22 (55)%
	n = 59	n = 152

Note

Figures are absolute numbers and percent for each row.

Of the nurses who viewed the pain scales positively, more assessed pain regularly than did those who viewed the scales negatively or neutrally (*P* = .003). Guidelines were available in the units according to 75% of respondents, whereas 16% were unsure and 9% stated that there were no guidelines in their units. Of the nurses who answered that they had guidelines in their units, 26% stated that the guidelines were followed, while 27% thought that they were not followed and 47% were unsure. Lack of time, ignorance, and negligence were some of the reasons offered as to why guidelines were not followed: “I believe that ignorance is the biggest reason why guidelines are not followed correctly. I think another reason is that it has not become a routine.” Lack of guideline implementation was also claimed to be a reason: “Ignorance. Bad understanding of how the guidelines should be followed. To write guidelines is easy but to implement them and strive for them is very hard.” Guidelines were also described as inaccessible.

## 4 DISCUSSION

The nurses in this study represent a majority of Swedish neonatal units. Most nurses thought that neonatal pain assessment was important and that they had sufficient knowledge of assessing pain in infants. Many nurses also stated that they used a pain scale regularly and viewed the use of the scale positively. Despite reporting adequate knowledge and regular use of pain assessment scales, difficulties with the assessments were highlighted. An impression that guidelines were not being followed and lack of pain assessment knowledge and routines were reasons mentioned for insufficient pain assessment. Some nurses also expressed doubts regarding the currently used pain scale in their unit.

In this study, some nurses were unsure about guideline compliance or believed that it was insufficient. Despite most nurses believing that the pain assessment of infants was important, only half of the respondents thought they had enough knowledge of pain and pain assessment. Nurses' attitudes toward and knowledge of neonatal pain affect the degree to which they assess pain.^16^ More knowledge resulted in a more positive attitude toward the pain assessment scale. This is in agreement with another study showing that nurses became more motivated and positive about pain assessment after receiving video education on the subject.^17^ Studies have further shown that there is a relationship between experience and knowledge, and that the nurses with more knowledge view pain assessment and treatment more positively.^16^

In the open answers, the nurses indicated that they were unsure whether the pain scale really measured what it was intended to measure and considered this being a problem. Blomqvist et al found that some nurses believed the pain assessment to be more adequate if done by their own clinical judgment than if strictly following a pain scale.^12^ If the pain is mistaken for something else and no intervention is made, there is a risk that the newborn infant's pain will be left untreated, with possible negative consequences for the infant.^5^ It is not unproblematic to assess whether an infant is in stress or in pain. Pain is stressful for the infant but not all stress is caused by pain. However, stress still needs to be treated with neuroprotective strategies to improve the infant's development.^18^

Unclear guidelines were also mentioned by the nurses as something that could impair pain assessment and pain management. If a guideline is implemented and validated correctly by a multidisciplinary team, it could enhance a positive attitude and motivation to follow it.^19^ It is reported by Pölkki et al^20^ that available guidelines increased the use of pain assessment compared to when nurses did not have instructions. This is however not unproblematic, and guidelines do not always increase pain management since there might still be a gap between knowledge and practice.^21^, ^22^ Pain management should always be performed in collaboration between nurses and physicians at the neonatal unit, but previous research has shown that nurses often have more knowledge about pain assessment than physicians.^12^ This could lead to sub‐optimal pain management since the physicians are responsible for prescription of pain medication if necessary, and need to trust the reported pain score.

The most frequently used pain scale according to the respondents was ALPS‐neo, a scale that has only been validated in one published study despite being frequently used clinically in Sweden.^11^, ^12^, ^23^

Some participating units did not have guidelines for pain assessment or pain scales implemented, according to the respondents. Several nurses who mentioned the lack of guidelines reported that pain assessment guidelines were about to be implemented and that a restart was about to happen in their unit. In a 2017 study comparing Swedish and Norwegian neonatal units, 87% of the Swedish head nurses confirmed that they used a validated pain scale and 85% documented pain regularly.^11^ In a study by Eriksson and Gradin,^10^ 83% of the units reported the regular use of pain assessment compared with 77% five years earlier. The use of pain assessment guidelines and of structured pain assessments has evidently increased over the years in Swedish neonatal units, although the present results indicate slightly less use than did earlier studies of such use reported by the head nurses in neonatal units. The literature is not unanimous that the introduction of guidelines is enough to change clinical praxis, as it takes time to implement them in daily care.^10^, ^11^, ^24^ Both international and Swedish guidelines recommend that all units should provide written guidelines for pain assessment and treatment to ensure the best possible treatment.^9^, ^25^, ^26^

The nurses in this study were asked if the pain assessment differed between different levels of care which they confirmed. It can be discussed that the guidelines also should define frequency and type of pain assessment in relation to level of the infant's care. In Sweden, most units provide family‐centered care and have high parental presence in all levels of care. Family‐centered care provides an opportunity to improve pain assessment and the nonpharmacological management by utilizing parental involvement.^12^ An interprofessional collaboration between parents and nurses could facilitate and contribute to an optimal pain management for the individual infant.^27^, ^28^

A Norwegian study highlighted lack of knowledge of pain and pain assessment as an obstacle to pain assessment; it also indicated that inadequate pain assessment could lead to missed pain treatment.^29^ In the present study, only 61% of the nurses believed that they had enough knowledge of neonatal pain, whereas nurses who had specialist nursing education rated their knowledge as higher and nurses with knowledge of the pain assessment scale viewed pain assessment more positively. The fact that only half of the Swedish neonatal nurses have enough knowledge of pain signals a knowledge gap that needs to be addressed.

### 4.1 Strength and limitations

Survey studies run the risk of not obtaining enough responses, and a low response rate reduces the representativeness of the population. The response rate in this study was acceptable, with 79% of the neonatal units responding and 61% (n = 231) of the surveys being returned.

One limitation of the study was the use of convenience sampling, in which respondents were selected by the head nurses of the neonatal units. There is a risk of sample bias if only nurses with a special interest in pain were chosen or perhaps only very experienced nurses. However, the ranges of age and of years worked indicate acceptable variation of the respondents. The optimal way to obtain representativeness would have been to invite all the staff members working at the units to participate. The method of using a selected proportion of nurses was used in a previous study, resulting in a good variety of respondents, so the sampling design was replicated here.^30^

A self‐constructed survey was used in this study, allowing us to create questions that relate to the specific aim of the study. Nevertheless, there are risks of nonobjective questions and of the authors' pre‐understanding being reflected in the questions. An expert in the field was involved in the construction of the survey. The validity of the survey could have been increased by using a panel of experts. A minor pilot study was performed in which the survey was administered to three persons working at a neonatal ward to discover misunderstandings and enhance content validity. We believe that a question about what can facilitate the use of adequate pain assessment would have strengthened the results.

## 5 CONCLUSION

Although nurses in general expressed a positive attitude toward pain assessment scales, this was not necessarily evident in their clinical practice. Lack of knowledge, available or accessible guidelines, or concerns regarding the validity of available pain scales seemed to limit their use.

### 5.1 Clinical implications

The aim of the study was to investigate nurses' perception, knowledge, and use of neonatal pain assessment in Swedish neonatal units. Raising and studying the subject can create awareness of the situation and illuminate weaknesses in daily clinical practice.

Despite extensive research into neonatal pain and the negative effects of untreated pain, nurses in Swedish neonatal units still do not appear to have sufficient knowledge of neonatal pain and pain assessment. There is a need for more education about neonatal pain, since this could result in more correct pain assessments and pain‐relieving treatments. There is also a need for clearer and more accessible guidelines, since the existing guidelines mentioned by the study respondents were not entirely followed because they were unclear and/or not implemented in the units.

### 5.2 Further research

Further research could replicate the present design but include a total population from the neonatal units to obtain a multidisciplinary view of neonatal pain management. To compare the staff nurses' views and experiences about pain assessment with the official policies of their units would also enrichen the picture. Conducting a qualitative study with the same aim would facilitate a deeper understanding of nurses' perceptions of neonatal pain management. An implementation study of education about neonatal pain and pain management could give directions as to how best to implement pain assessment knowledge in neonatal units. More validation of existing pain assessment scales is needed to ensure that these scales are measuring pain correctly.

## ETHICAL APPROVAL AND CONSENT TO PARTICIPATE

At the time of the study, the authors were higher‐level students and, according to the Swedish Ethical Review Act (SFS 2003:460), no ethics approval is needed for survey studies of clinicians. Ethical matters were considered throughout the study according to the Helsinki Declaration.^31^ Informed consent was obtained, in that respondents were informed that participation was voluntary. By completing the survey, the respondents gave their consent to participate.

## CONFLICT OF INTEREST

There are no conflicts of interest to disclose.

## Supporting information

Appendix S1
