# Supplementary material for: Nurses' perception, knowledge, and use of neonatal pain assessment
Source: Paediatr Neonatal Pain. 2021 May 7;3(2):59–65. doi: 10.1002/pne2.12050 (PMC8975217; doi:10.1002/pne2.12050)
Supplement: Supplementary file 1 — Appendix S1 [file PNE2-3-59-s001.docx]

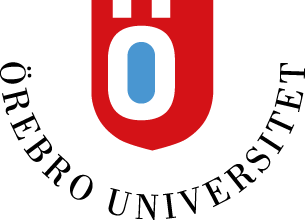


**Survey about neonatal pain assessment**

**1. Gender**

- **Women**
- **Man**
- **Other**

**2. Age**

**3. Number of years working as a nurse in neonatal care:**

**4. Do you have any specialist education? If yes please specify:**

**5. Choose the level of care that best represents your unit**

| - **Level 1** | **Care of infants born in gestational week 35-36 who are physiological stable, and stabilization of infants born <35 weeks and sick infants until transfer to another hospital with higher level of care is possible.** |
| --- | --- |
| - **Level 2** | **Specialized care of infants born ≥ gv32 or birthweight ≥1500gram, with possibility of short-time ventilator treatment or CPAP.** |
| - **Level 3A** | **Subspecialized intensive care of infants born ≥28 gestational weeks or birthweight ≥ 1000gram with possibility of ventilator treatment.** |
| - **Level 3B** | **Subspecialized intensive care of infants born <28 gestational weeks or weighs <1000gram with possibility of advanced respiratory support and access to pediatric surgery.** |
| - **Level 3C** | **Same as 3B and ECMO and surgery of complex congenital heart defects.** |
| - **Other** | **If the unit does not fit into any of the above mentioned levels of care describe which type of care that is administered at the unit and for whom.** |

**Attitudes and knowledge about pain assessment**

**6. How important do you think neonatal pain assessment is?**

Not important at all 1 2 3 4 5 Very important

**7. How much knowledge do you have of neonatal pain assessment?**

Inadequate 1 2 3 4 5 More than good enough

**8. How did you acquire your knowledge about pain in infants?**

**9. To what extent do you think the care of the infant is improved by pain assessment ?**

Not at all 1 2 3 4 5 Great improvement

**The use of a validated pain scale**

**10. Which pain scale/s is used in your unit?**

**11. What are our attitude towards the pain scale that is used in your unit?**

Very negative 1 2 3 4 5 Very positive

**12. Do you use a pain scale regularly in your work?**

- Yes
- No

**13. If yes, how often?**

- Several times per shift
- Once per shift
- A couple of times a week
- A couple of times a month

**14.** **Do you think you have enough knowledge in order to use a pain scale correctly?**

- Yes
- No
- Uncertain

**15. Do you perceive differences in the regularity of pain assessments between infants who are in need of intensive care and infants who are cared for in intermediate care/family care?**

- Yes
- No
- Uncertain

**16. What type of measure do you feel best demonstrates pain in the infants?**

- A validated pain scale
- My clinical sense
- Physiological parameters
- A combination of the above
- Something else:

**17. Do you think there are problems with pain assessment?**

- Yes
- No

**18. If the answer is yes, what problems do you experience?**

**19. Are there guidelines for pain assessment and treatment at your unit?**

- Yes
- No
- Unsure

**20. Are these guidelines followed properly?**

- Yes
- No

**21. If no, what are the reasons the guidelines are not followed?**

**22. If an infant show signs of pain during the pain assessment, to what extent do you think the infants receives pain treatment according to the guidelines?**

To a small extent 1 2 3 4 5 To a large extent
